# Entecavir and lamivudine therapy for severe acute chronic hepatitis B

**DOI:** 10.3892/etm.2012.850

**Published:** 2012-12-05

**Authors:** CHANGHONG LIU, JING YE, HONGYU JIA, MEIJUAN ZHANG, HONGFENG HAN, FENGZHE CHEN, CONGKANG CHEN

**Affiliations:** 1 Department of Gastroenterology, Shandong Provincial Qianfoshan Hospital; Zhejiang University, Zhejiang;; 2 Department of Infectious Diseases, Lishui Central Hospital; Zhejiang University, Zhejiang;; 3 Department of Infectious Diseases, The First Affiliated Hospital of the College of Medicine, Zhejiang University, Zhejiang;; 4 Department of Gastroenterology, Luoyang Central Hospital, Henan;; 5 Department of Infectious Diseases, Qilu Hospital of Shandong University, Jinan;; 6 Department of Internal Medicine, Beilun District Hospital of Chinese Medicine, Ningbo, P.R. China

**Keywords:** entecavir, lamivudine, severe acute exacerbation, hepatitis B virus

## Abstract

Severe acute exacerbation in patients with chronic hepatitis B is a unique clinical presentation that results in high mortality. To compare the efficacy of 4 weeks of entecavir or lamivudine therapy in patients with severe acute exacerbation caused by chronic hepatitis B, 65 patients were retrospectively analyzed. The groups received 0.5 mg entecavir (group A) or 100 mg lamivudine (group B) daily. After 4 weeks of treatment, virological and biochemical responses and the deterioration rates were determined. The average age and alanine aminotransferase (ALT), bilirubin, albumin, creatinine (Cr), and HBV DNA levels were similar in groups A and B prior to treatment, but the prothrombin time (PT) was shorter in group A. Following treatment, ALT and bilirubin levels were improved while there were no significant changes in PT, albumin or Cr levels at week 4 in group B. Only ALT was markedly upregulated following treatment in group A. There were no significant differences between the virological and biochemical responses or the deterioration rates of the two groups. These results suggested that short-term treatment with lamivudine markedly alleviated the increased bilirubin (TB) levels in patients with severe acute exacerbation of chronic hepatitis B and that there was no significant difference in the deterioration rate between patients treated by the two types of medication.

## Introduction

Chronic hepatitis B virus (HBV) infection is a public health concern worldwide and is responsible for 1 million deaths annually. The virus significantly increases the risk of hepatitis, cirrhosis and hepatocellular carcinoma (1). Severe acute exacerbation is a unique presentation of chronic HBV infection which is characterized by high alanine aminotransferase (ALT) levels accompanied by jaundice and hepatic decompensation (2). Deterioration leads to acute-on-chronic liver failure and mortality (3).

Lamivudine was the first registered oral antiviral drug and has been widely used due to its efficacy during short-term administration (4). Outcomes regarding the use of lamivudine therapy for patients with severe acute exacerbation of chronic hepatitis B have been reported (5–7). However, the therapy is limited by the high risk of virological breakthrough and drug resistance, which has been observed in up to 76% of patients that have been treated for ≥5 years (8). A better drug with a lower risk of drug resistance is required. Entecavir is a new and potent drug that works against HBV by selective inhibition of HBV DNA polymerase. With regard to safety, entecavir is similar to lamivudine although it is superior in tolerability (9–11). However, few studies have observed the outcome of entecavir for patients with severe acute exacerbation of chronic hepatitis. A recent study showed that entecavir treatment was associated with increased short-term mortality in patients with severe acute exacerbation of chronic hepatitis B, particularly within 30 days following treatment (12).

The present study retrospectively compared the effect of entecavir and lamivudine in the short-term treatment (30 days) of patients with severe acute exacerbation of chronic hepatitis.

## Patients and methods

### Patients

A total of 65 patients who were consecutively admitted to the First Affiliated Hospital of the College of Medicine, Zhejiang University, China, between May, 2008 and May, 2011 with spontaneous severe acute exacerbation of chronic hepatitis B, were retrospectively studied. Severe acute exacerbation of chronic hepatitis B was defined as an elevation of ALT to a level ≥10-fold higher than the upper limit of normal (ULN, 40 IU/l) and an elevation of bilirubin to a level ≥3-fold higher than the ULN (21 *μ*mol/l). All 65 patients had been recorded as positive for the hepatitis B surface antigen (HBsAg) for >6 months. Patients coinfected with hepatitis A, C and E viruses were excluded by serological assays. An abdominal ultrasound was performed at admission to exclude cases of hepatocellular carcinoma and biliary obstruction. There were no patients with hepatic encephalopathy, ascites or abnormal renal function tests at baseline. Patients who received immunosuppressants or systemic corticosteroids were also excluded. Cirrhosis in patients was defined by an ultrasound revealing a coarse liver echotexture with nodularity and small liver size and the presence of portal hypertension (e.g., ascites, splenomegaly and varices). Prior written and informed consent was obtained from each patient and the study was approved by the ethics review board of Shandong University.

The eligible subjects were divided into two groups. Group A consisted of 31 patients who received 0.5 mg entecavir daily and group B consisted of the remaining 34 patients who were administered with 100 mg lamivudine daily. The levels of serum ALT, bilirubin, PT, albumin, creatinine (Cr) and HBV DNA titer were analyzed at week 4 following the treatment. The deterioration rate, defined as the rate of disease progression in a patient taken from the date when the first dose of lamivudine or entecavir was administered to the date of acute-on-chronic liver failure or mortality, was also calculated.

### Real-time quantitative PCR

HBV DNA was measured by real-time polymerase chain reaction on a LightCycler 2.0 (Roche Diagnostics, Indianapolis, IN, USA). The fluorescence detection kit and probes were purchased from Piji Biological Engineering Co., Ltd. (Shenzhen, China). The detection limit of the HBV DNA was 1,000 copies/ml and the level of negative HBV DNA was set as 500 copies/ml.

### Statistical analysis

Statistical analysis was performed by SPSS software (version 13). In the present study, continuous variables were expressed as the mean ± standard deviation (SD) or median (range) values. The level of HBV DNA was logarithmically transformed to a normal distribution for analysis. The significance of the differences between the results of Levene’s homogeneity examination of the normalized continuous variables was determined by the Student’s t-test. The significance of the differences between the categorical variables was analyzed by the Chi-square test. Non-conformity in the baseline between the two groups was eliminated by covariance analysis of the prothrombin time (PT). P<0.05 was considered to indicate a statistically significant difference.

## Results

### PT was shorter in group A patients prior to treatment

To determine whether the baseline conditions of subjects in groups A and B (31 and 34 patients ingesting 0.5 mg entecavir and 100 mg lamivudine daily, respectively) were the same prior to the treatment, biochemical markers were analyzed. No significant differences were detected in gender or age between the two groups (P=0.309 and 0.524, respectively) (Table I). The baseline levels of ALT, bilirubin, albumin and Cr at the time antiviral therapy was commenced were similar between the two groups. No difference was observed between the levels of HBV DNA (P=0.077), however, PT was shorter in group A compared with group B (P=0.021).

### Responses following the 4-week treatment

To study the changes in patient responses prior to and following treatment, levels of ALT, bilirubin, albumin, Cr, HBV DNA and PT were analyzed. Following the 4-week treatment of enticavir, patients in group A showed a marked downregulation of ALT (P=0.000, Table II), with a significant downregulation of HBV DNA levels (P=0.001). However, the levels of bilirubin, albumin, Cr and PT remained almost unchanged. In group B, the patients treated with lamivudine for 4 weeks demonstrated improved levels of ALT and bilirubin (P=0.000 and P=0.005, respectively) compared with those observed prior to treatment. HBV DNA levels were also markedly decreased (P=0.000).

### Comparison of the two groups following treatment by enticavir or lamivudine

The present study investigated the difference in the efficiency of enticavir and lamivudine treatment. There were no significant differences in PT or in the levels of ALT, TB, ALB and Cr between the two groups following the 4-week treatment (Table III). Moreover, the levels of HBV DNA (l g) were unchanged between the two groups (P=0.213).

Acute chronic liver failure (ACLF) developed in 2 patients (6.45%) in group A, while 2 patients (5.88%) in group B deteriorated into hepatic failure, with one patient undergoing liver transplantation. Therefore the deterioration rates of the two groups were similar (P=0.385).

## Discussion

The present study is a retrospective analysis of patients enrolled between May 2008 and May 2011. Previous studies have reported that entecavir is as safe as Lamivudine, but that it is able to inhibit the replication of HBV faster and more efficiently, leading to improved clinical outcomes when used in patients with chronic hepatitis B (13,14). Although severe acute exacerbation in patients with chronic hepatitis B characterized by extremely high ALT levels accompanied by jaundice and hepatic decompensation is difficult to cure, few studies have focused on the efficacy of entecavir in patients with this condition. Wong *et al* showed that while entecavir was the better inhibitor of hepatitis B, the mortality rate within 30 days was significantly higher than when using lamivudine (p=0.028) (12). In the present study, every indicator, including PT, exhibited no evident differences between the groups treated either with entecavir or lamivudine for 4 weeks. The deterioration rates between the two groups were also the same. These results demonstrated that the efficacy of lamivudine and entecavir was similar in the treatment of patients with severe acute exacerbation of chronic hepatitis B.

The study of Wong *et al*(12) also demonstrated that entecavir was able to lead to a more rapid decrease in HBV DNA compared with lamivudine and that this decrease may be associated with the high mortality rate demonstrated with entecavir treatment. However, in the present study, no differences in the levels of HBV DNA between patients treated with entecavir and lamivudine were detected. Further observation is required to investigate the correlation between HBV DNA levels and mortality. There are several factors that may affect these results. Firstly, the patients selected in the present study had severe acute exacerbation of chronic hepatitis B and the typical presentation of severe acute exacerbation is a short onset of jaundice and an extremely high ALT level. Moreover, these patients may have strong immune scavenging and e-antigen negative conversion abilities (2). Therefore within a short time, entecavir and lamivudine may exhibit good outcomes. Secondly, the analyses on genotype and other influencing factors were not accessible as the present study was only a retrospective analysis.

The present study identified that besides being a virological indicator, lamivudine was able to significantly reduce the levels of ALT and bilirubin, while entecavir was only able to decrease the ALT level. These results showed that lamivudine was superior in decreasing bilirubin levels as compared to entecavir, as observed by Wong *et al*(12), but that bilirubin levels were thought to be associated with the higher morbidity of entecavir.

The main purpose of the present study was not to observe the short-term effects of entecavir treatment, but to prove whether entecavir would significantly increase the short-term deterioration rate and thus cause its long-term application to be restricted. Current studies have yet to demonstrate the manner in which the anti-viral therapy may improve the short-term prognosis of patients with severe acute exacerbation of chronic hepatitis B (15,16). Certain investigators believed that short-term mortality mainly depended on the degree of hepatic necrosis but not the viral load (16,17). Treatment with nucleo-side analogues, including lamivudine, was recommended for patients with severe acute exacerbation of chronic hepatitis B mainly in order that they be provided with long-term therapeutic benefits (16). However, long-term lamivudine treatment caused severe drug-resistance (8). Current evidence does not appear to support adefovir salvage therapy either (18,19). Entecavir was not recommended to patients with severe acute exacerbation of chronic hepatitis B based on the studies by Wong *et al*(12) which demonstrated a marked elevation of short-term mortality, although the mortality rate had no significant differences between 30 days and 48 weeks (P=0.14). In the present study, entecavir did not show any superiority over lamivudine, particularly in its ability to reduce bilirubin levels. However, entecavir also did not lead to the elevation of short-term mortality rates. Consequently further investigation is required to confirm that entecavir is potentially a better choice for the anti-viral therapy in patients with severe acute exacerbation of chronic hepatitis.

## Figures and Tables

**Table I.** Baseline characteristics of patients with severe acute exacerbation of chronic hepatitis B prior to entecavir or lamivudine treatment.

Characteristics	Group A	Group B	P-value
Gender (M:F)	28:3	27:7	0.309
Age (mean)	41.40±11.54	43.47±11.97	0.524
PT (sec)	14.03±2.38	15.38±2.19	0.021
ALT (IU/l)	1056.23±725.88	903.94±856.28	0.444
TB (*μ*mol/l)	206.70±112.70	248.43±141.68	0.196
ALB (g/l)	36.33±5.97	37.27±5.38	0.506
DNA (log copies/ml)	6.23±1.88	7.04±1.68	0.077
Cr (*μ*mol/l)	65.04±13.85	73.33±23.70	0.220

A total of 31 patients received 0.5 mg entecavir daily and were designated as group A, while 34 patients were administered with 100 mg lamivudine daily and were designated as group B. PT, prothrombin time; ALT, alanine aminotransferase; TB, total bilirubin; ALB, albumin; Cr, creatinine.

**Table II.** Clinical outcomes of entecavir and lamivudine treatment in patients with severe acute exacerbation of chronic hepatitis B.

	Entecavir				Lamivudine			
Marker	Prior to treatment	After treatment	t	P-value	Prior to treatment	After treatment	t	P-value
PT (sec)	14.03±2.38	13.52±3.60	0.543	0.592	15.38±2.19	16.15±12.89	−0.291	0.773
ALT (IU/l)	1056.23±725.88	72.27±46.19	6.749	0.000	903.94±856.28	95.39±29.72	6.078	0.000
TB (*μ*mol/l)	206.70±112.70	160.46±189.47	1.271	0.214	248.43±141.68	165.67±185.44	2.983	0.005
ALB (g/l)	36.33±5.97	36.85±4.86	−0.635	0.531	37.27±5.38	38.71±6.33	−1.225	0.229
Cr (*μ*mol/l)	68 (58,77)	68.5 (39,425)	0.793	0.428	60 (54,71)	62 (36,173)	1.412	0.158
DNA (log copies/ml)	6.48 (5.05,8.44)	2.70 (2.70,4.24)	3.233	0.001	6.18 (4.75,7.68)	2.70 (2.70,4.48)	4.356	0.000

Differences in PT, ALT, TB and ALB were analyzed by paired t-tests and differences in Cr and DNA (l g) levels were analyzed by Wilcoxon signed-rank tests. The levels of PT, ALT, TB and ALB were presented as the mean ± SD and the levels of DNA and Cr were presented as median (ICQ) values. PT, prothrombin time; ALT, alanine aminotransferase; TB, total bilirubin; ALB, albumin; Cr, creatinine.

**Table III.** Comparison of the biochemical markers following a 4-week treatment of entecavir or lamivudine.

Marker	Entecavir	Lamivudine	P-value
PT (sec)	13.52±3.60	16.15±12.89	0.143
ALT (IU/l)	72.27±46.19	95.39±29.72	0.476
TB (*μ*mol/l)	160.46±189.47	165.67±185.44	0.912
ALB (g/l)	36.85±4.86	38.71±6.33	0.200
DNA (l g)	2.70 (2.70, 4.48)	4.00 (2.70, 4.90)	0.213
Cr (*μ*mol/l)	68.10±24.47	77.62±38.11	0.327

Levels of PT, ALT, TB and ALB were presented as the mean ± SD. The levels of DNA and Cr were presented as median (ICQ) values. PT, prothrombin time; ALT, alanine aminotransferase; TB, total bilirubin; ALB, albumin; Cr, creatinine.
